# Effect of the PHY Domain on the Photoisomerization Step of the Forward P_r_→P_fr_ Conversion of a Knotless Phytochrome

**DOI:** 10.1002/chem.202003138

**Published:** 2020-11-27

**Authors:** Tobias Fischer, Qianzhao Xu, Kai‐Hong Zhao, Wolfgang Gärtner, Chavdar Slavov, Josef Wachtveitl

**Affiliations:** ^1^ Institute of Physical and Theoretical Chemistry Goethe University Frankfurt am Main Max-von-Laue Straße 7 60438 Frankfurt Germany; ^2^ Institute of Analytical Chemistry University of Leipzig Linnéstr. 3 04103 Leipzig Germany; ^3^ Key State Laboratory of Agriculture Microbiology Huazhong Agriculture University Wuhan Shizishan Street, Hongshan District Wuhan 430070 P. R. China

**Keywords:** bilin-binding photoreceptors, photochemistry, photoisomerization, time-resolved spectroscopy

## Abstract

Phytochrome photoreceptors operate via photoisomerization of a bound bilin chromophore. Their typical architecture consists of GAF, PAS and PHY domains. Knotless phytochromes lack the PAS domain, while retaining photoconversion abilities, with some being able to photoconvert with just the GAF domain. Therefore, we investigated the ultrafast photoisomerization of the P_r_ state of a knotless phytochrome to reveal the effect of the PHY domain and its “tongue” region on the transduction of the light signal. We show that the PHY domain does not affect the initial conformational dynamics of the chromophore. However, it significantly accelerates the consecutively induced reorganizational dynamics of the protein, necessary for the progression of the photoisomerization. Consequently, the PHY domain keeps the bilin and its binding pocket in a more reactive conformation, which decreases the extent of protein reorganization required for the chromophore isomerization. Thereby, less energy is lost along nonproductive reaction pathways, resulting in increased efficiency.

## Graphical abstract

**More reactive, more efficient**: The influence of the PHY domain on the photoconversion of a knotless phytochrome was investigated. The interaction of its “tongue” region with the GAF domain keeps the bilin chromophore and the binding pocket in a more reactive conformation. This results in accelerated reorganization of the protein environment necessary to accommodate the photoisomerization of the chromophore and, in effect, in an increased overall quantum efficiency.

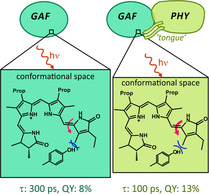

## Introduction

**Scheme 1:**
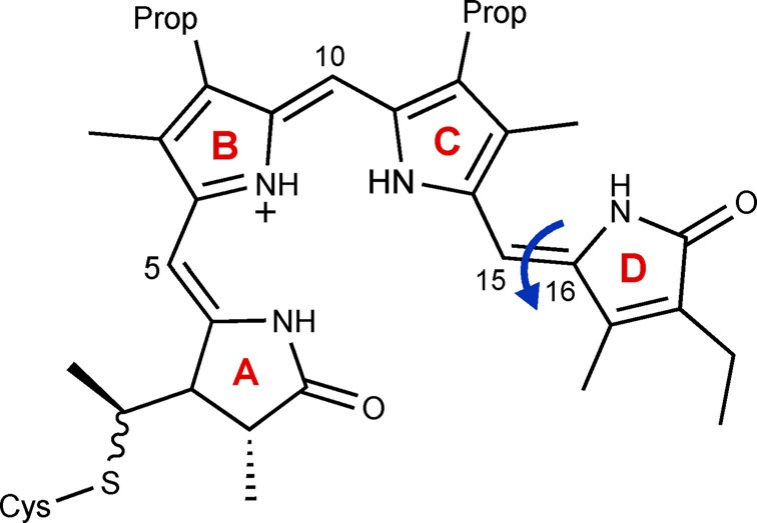
Chemical structure of the phycocyanobilin (PCB) chromophore. The arrow indicates the photoisomerization of the C_15_=C_16_ double bond and the counterclockwise rotation of ring D.^[8]^

Phytochromes are bilin‐binding photoreceptors that regulate various biologically relevant processes (e.g., photosynthesis, morphogenesis, phototaxis, and photoprotection).[^1^, ^2^] They function via a light‐induced transformation between a thermostable parental state and a photoproduct state. The transformation is triggered by a *Z* ⇄ *E* photoisomerization of the C_15_=C_16_ double bond and a subsequent rotation of the d‐ring of the embedded bilin chromophore (Scheme 1).

**Figure 1:**
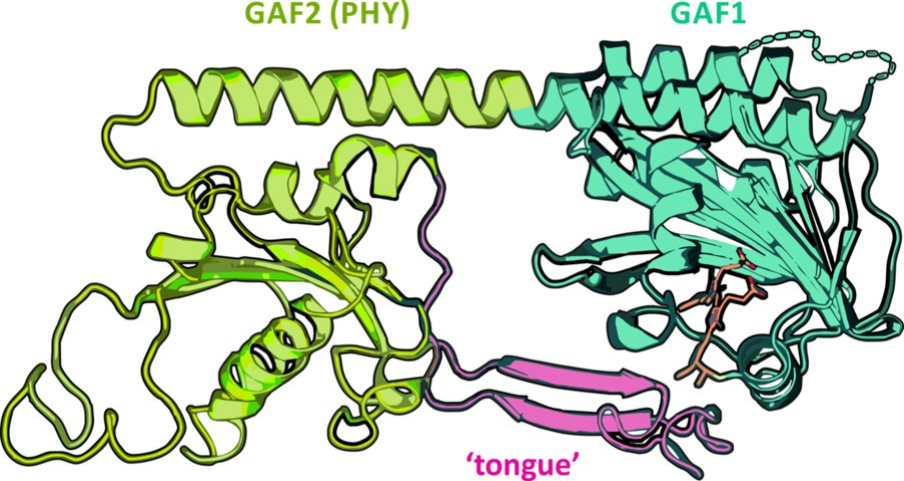
Schematic representation of the structural homology model of the P_r_ state of the All2699g1g2 construct.^[32]^ The structure of the GAF1 domain is based on the crystal structure of the sole GAF1 module (PDB ID 6OZA), while the GAF2 domain was modelled based on the crystal structure of the structurally similar Cph2^[32]^ (PDB ID 4BWI). The PCB chromophore (orange) embedded in the GAF1 domain (green) is in close interaction with a tongue‐like protrusion (pink) from the GAF2 domain (yellow), which also shields the PCB from the solvent.

Phytochromes consist of chromophore‐binding GAF domains, PAS and PHY domains (Figure 1).[^1^, ^2^] The PHY domain forms an antiparallel β‐sheet (“tongue”) which interacts with the chromophore embedded in the GAF domain, and the PAS and the GAF domains form a figure‐eight knot.[^1^, ^2^] A complete PAS‐GAF‐PHY array is required for photoconversion in canonical phytochromes (e.g., PhyA, Cph1, Agp1),[^3^, ^4^, ^5^] while knotless phytochromes (e.g., Cph2)[^6^, ^7^] lack the PAS domain.[^1^, ^2^]

Interestingly, single GAF domains of cyanobacteriochromes (CBCRs)^[9]^ and some knotless phytochromes[^8^, ^10^] preserve their photoconversion capability, which makes them attractive for biotechnological applications.[^11^, ^12^] The parental and the photoproduct states of canonical and knotless phytochromes are red‐light (P_r_) and far‐red‐light (P_fr_) absorbing, respectively.^[5]^ The photoisomerization reaction and the primary photoproduct (Lumi‐R) formation in the forward (P_r_→P_fr_) photoconversion of these phytochromes proceeds with a lifetime of tens[^13^, ^14^, ^15^, ^16^, ^17^, ^18^, ^19^, ^20^] to hundreds[^21^, ^22^, ^23^, ^24^, ^25^] of picoseconds. Photoisomerization in other molecular systems (e.g., azobenzenes[^26^, ^27^] and rhodopsins[^28^, ^29^, ^30^]) is typically ultrafast, which raises the question about the origin of the remarkably slow photoisomerization rates in phytochromes. Recently, we could show that the excited state decay kinetics in a single GAF domain (g1) derived from a knotless phytochrome (All2699g1g2 from *Nostoc* sp. PCC7120) is strongly distributed, and we assigned this behavior to conformational changes in the bilin‐binding pocket that control the photoisomerization of the chromophore.^[8]^ Here, we report on the forward (P_r_→P_fr_) photoisomerization dynamics of the complete knotless phytochrome All2699g1g2 (structurally similar to Cph2).[^31^, ^32^] The homology model of All2699g1g2^[32]^ (Figure 1) shows that the “tongue” region of the g2 domain interacts with the chromophore bound to g1, just like the PHY domains of canonical phytochromes. Therefore, the g1g2 construct provides a unique opportunity to directly evaluate the role of the “tongue” and thereby of the protein matrix in the photoisomerization of phytochromes. Furthermore, our results give insight into the photochemistry of knotless phytochromes, as their ultrafast dynamics has not been studied previously.

## Results and Discussion

### Photochromism of All2699 g1 g2

**Figure 2:**
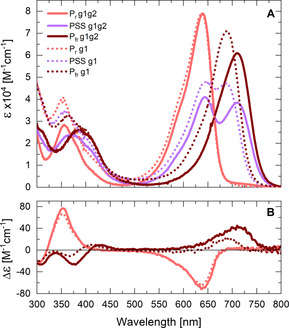
A) Stationary absorption spectra of the P_r_ and P_fr_ forms of g1g2 (solid lines) and g1^[8]^ (dotted lines). The pure P_fr_ spectrum was obtained by conservative subtraction of 38 % of the pure P_r_ spectrum from the PSS spectrum, followed by multiplication with a factor of 1.61 to yield the spectrum for the fully converted system. The extinction coefficient of P_r_ is ∼79 000 m ^−1^ cm^−1^ (similar to g1^[33]^), while the extinction coefficient of P_fr_ is ∼61000 m ^−1^ cm^−1^. B) CD spectra of the P_r_ and P_fr_ states of g1g2 (solid lines) and g1^[8]^ (dotted lines). The pure P_fr_ CD spectrum was derived from the PSS CD as described in A).

The P_r_ form of g1g2 has an absorption maximum at 637 nm, which is similar to that in g1,[^8^, ^33^] while the P_fr_ form is significantly red shifted (by 73 nm) to 710 nm (689 nm in g1;[^8^, ^33^] Figure 2 A). Thus, the observed spectral shift of P_fr_ appears to be induced by interactions of the PCB chromophore with the “tongue” region of the g2 domain in g1g2.[^32^, ^34^] Interestingly, we find that the quantum yield (QY) of the P_r_→P_fr_ transition is increased from ∼8 % to ∼13 % (a similar QY is observed in the related Cph2^[35]^). Hence, it follows that while the presence of the g2 domain does not directly affect the spectral properties of the P_r_ form it does affect its photochemistry.

The circular dichroism (CD) spectra of both proteins (g1g2 vs. g1) have similar shape and undergo a sign inversion of the Q‐band CD signal upon P_r_↔P_fr_ switching (Figure 2 B).[^8^, ^33^] The Q‐band CD signals exhibit opposite signs for the different states, negative for P_r_ and positive for P_fr_. The P_fr_ signal of g1g2 is further red shifted and shows an increased extinction coefficient.

The signs of these signals indicate the orientation of the peripheral rings A and D with respect to the co‐plane of rings B and C.[^8^, ^36^] This overall orientation appears unaffected by the presence of the “tongue”, which is in line with the slight changes in the dihedral angle of rings A and D observed by NMR.^[32]^ In comparison, other phytochromes like Cph1^[37]^ and Cph2^[35]^ exhibit a similar sign change of the Q‐band, while most CBCRs and bacteriophytochromes show no sign change upon switching.[^36^, ^38^, ^39^, ^40^]

### Ultrafast dynamics of P_r_^*^ and formation of Lumi‐R

**Figure 3:**
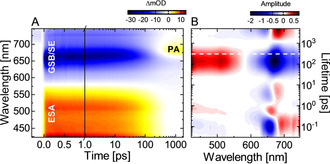
A) TA data from the forward, P_r_→P_fr_, dynamics of g1g2 after 635 nm excitation. B) Corresponding lifetime density map (LDM) obtained from the lifetime distribution analysis of the TA data. The reading of the LDMs is as for a decay‐associated spectrum from global lifetime analysis: i) positive (red) amplitudes account for decay of excited state and product absorption (ESA, PA) or rise of ground state bleach and stimulated emission (GSB and SE); ii) negative (blue) amplitudes account for rise of absorption (ESA, PA) or decay of GSB and SE. The white dashed line indicates the center of the lifetime distribution of the P_r_^*^ decay of g1.^[8]^

The role of the protein environment on the ultrafast photoisomerization dynamics of the PCB chromophore was investigated by femtosecond transient absorption (TA) measurements on g1g2 as compared with the single‐domain g1.^[8]^ The TA data of g1g2 show three main features (Figure 3 A): i) a broad positive signal below 575 nm which can be assigned to excited state absorption (ESA), ii) a negative signal above 575 nm due to ground state bleach (GSB) and stimulated emission (SE), and iii) a positive photoproduct absorption (PA) appearing at later times at 670 nm associated with the formation of the primary photoproduct (Lumi‐R). The rise of the Lumi‐R signal coincides with the decay of the ESA, GSB and SE on the timescale of 100 to 400 ps.

The lifetime distribution analysis of the experimental data (see Supporting Information and Ref. [41] for methodology) gives further insight into the early ES dynamics of P_r_ (Figure 3 B). The positive‐ (>690 nm) and the negative‐amplitude (<690 nm) distributions with a lifetime of 100 fs can be assigned to a red shift of the SE and therefore to the departure of the ES wavepacket from the Franck‐Condon (FC) region. The lifetime distributions between 1 ps and 10 ps are located at the overlap of the steep edges of the GSB and SE, making this region very sensitive to slight spectral changes.

Because there is no substantial decay of the ES on this timescale, we assign these distributions to dynamics on the ES potential energy surface. Based on the spectral position of the ESA, GSB and SE signals, the broad lifetime distributions (stretching from 30 ps to 1 ns) with positive (420–575 nm) and negative (600–740 nm) amplitudes can be attributed to the simultaneous decay of these signals, and thus to the decay of P_r_^*^.

Compared with the positive‐amplitude distribution representing the decay of the ESA signal, the negative‐amplitude distribution, especially at 675 nm, appears stretched in lifetime. This can be explained by an overlaid additional negative‐amplitude distribution describing the rise of the primary photoproduct Lumi‐R commencing with the ES decay. On the scale longer than 1 ns, the negative and positive‐amplitude lifetime distributions correspond to the non‐decaying GSB and Lumi‐R signals.

### Distributed character of the P_r_^*^ photoisomerization kinetics

**Figure 4:**
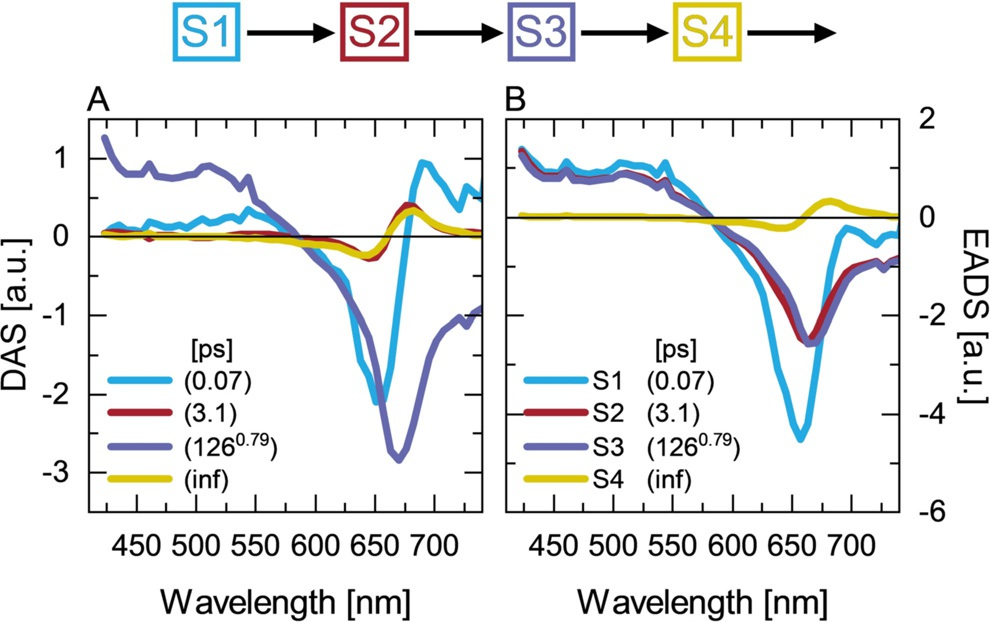
Analysis of the experimental data from the P_r_→P_fr_ dynamics of g1g2 after 635 nm excitation using a sequential kinetic scheme. The kinetic model fitting results in the so‐called evolution‐associated difference spectra (EADS) and decay associated spectra (DAS): A) DAS and B) EADS from fitting a sequential scheme with four states, with the third state (S3) being modeled with a stretched exponent with β=0.79.

Previously, we showed that the P_r_^*^ decay kinetics in the single‐domain g1 is described by broad and structureless lifetime distributions (see Figure S2B and discussion in^[8]^) and can be modelled well using a stretched exponential function,[^42^, ^43^] thereby avoiding introduction of unnecessary kinetic components. The lifetime distributions describing the P_r_^*^ decay of g1g2 (from 30 ps to 1 ns in Figure 3 B) are similarly broad and structureless, thus we followed our previous approach and analyzed the TA data using a four‐state model in which one of the states is modelled by a stretched exponent (Figure 4). This model yields an excellent fit of the data without additional kinetic components (compare the case of the five‐state model (Figure S5)). Stretched exponentials are used to model distributed kinetics occurring in constrained environments[^44^, ^45^, ^46^] and here underline the importance of the protein in the isomerization kinetics of the PCB chromophore.

The evolutionary associated difference spectra (EADS) of the first three states (Figure 4 B) clearly show that at early times no significant change in the amplitude of the ESA occurs, reaffirming the conclusion that the sub‐20 ps dynamics of P_r_^*^ is not associated with ES decay. Therefore, similarly to the single GAF domains All2699g1^[8]^ and Slr1393g3,^[47]^ the EADS of S2 and S3 of g1g2 show only a minor spectral shift in the GSB and SE overlap area, indicating that the ∼3 ps component (Figure 4) is due to ES dynamics of the chromophore. The P_r_^*^ relaxation and the associated PCB photoisomerization occur on the 100 ps timescale from state S3 after overcoming a barrier on the ES potential energy surface.^[8]^ This state is modeled as a stretched exponent, showing that the P_r_^*^ decay of g1g2 follows a distributed type kinetics.

Recently, it was reported that in g1 and in g1g2 there exists a broad distribution of ground state subconformations that rapidly interconvert in solution.[^32^, ^48^] These subconformations could partially contribute to the observed distributed character of the P_r_^*^ decay kinetics. However, their rapid interconversion denotes that they are separated by low energetic barriers, and thus cannot explain the large excited state barrier that determines the relatively slow P_r_^*^ decay kinetics (100 ps timescale).^[8]^ Such a barrier can be overcome only via dynamic reorganization of the system, which in turn provides the dominant contribution to the distributed kinetics of P_r_^*^ decay. Therefore, our results point to a more dynamic picture of the kinetics in knotless phytochromes. In contrast, distinct ES decay components, including such on the sub‐50 ps timescale, have been reported for other phytochromes (e.g., Cph1, PhyA) and CBCRs, and were discussed in the framework of static ground state heterogeneity of the P_r_ form.[^15^, ^18^, ^20^, ^38^, ^49^, ^50^]

### Comparison of the ultrafast dynamics of g1 g2 and g1: the effect of the g2 (PHY) domain

**Figure 5:**
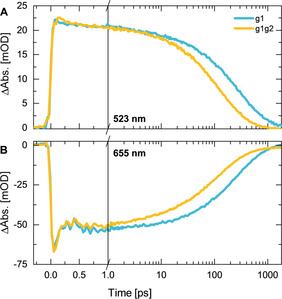
Comparison of the transient absorption decays of g1g2 (orange) and g1^[8]^ (cyan) at selected wavelengths within A) the ESA (523 nm), and B) the GSB/SE (655 nm) spectral regions. The transient decays were measured after 635 nm excitation of the P_r_ form.

The comparison of the ultrafast dynamics of the g1g2 construct with the dynamics of the sole GAF domain g1^[8]^ provides a direct assessment of the effect of the g2 (PHY) domain. Strikingly, the early dynamics of both proteins are identical and even the coherent oscillations observed in the SE region up to ∼2 ps (Figure 5 B) match in frequency and phase.

The similarity in the g1g2 and g1 early kinetics is in agreement with the lack of an immediate effect of the PHY domain on the steady state properties of the P_r_ form (Figure 2), and thus further supports the conclusion that the primary dynamics are indeed due to conformational changes in the PCB chromophore. Only at later times, the P_r_^*^ kinetics of g1g2 deviates from that of g1. Figure 5 clearly shows that in the g1g2 construct the ES decay and the formation of the primary photoproduct Lumi‐R are accelerated relative to g1. This effect is also illustrated by the corresponding LDMs (Figure S2). Considering the time range up to 10 ps, the lifetime distribution structure remains the same for both proteins, while the later lifetime distributions (from 30 ps to 1 ns) describing the ES decay are shifted to shorter lifetimes in the case of g1g2 (for comparison, the center lifetime of the corresponding g1 distribution is indicated by a white dashed line in the LDM of g1g2, Figure 3 B). Therefore, the direct comparison of the P_r_^*^ kinetics of g1g2 and g1 reveals the impact of the g2 (PHY) domain and categorically demonstrates the critical role of the protein environment on the photoisomerization step of the PCB chromophore.

### Mechanistic model

**Figure 6:**
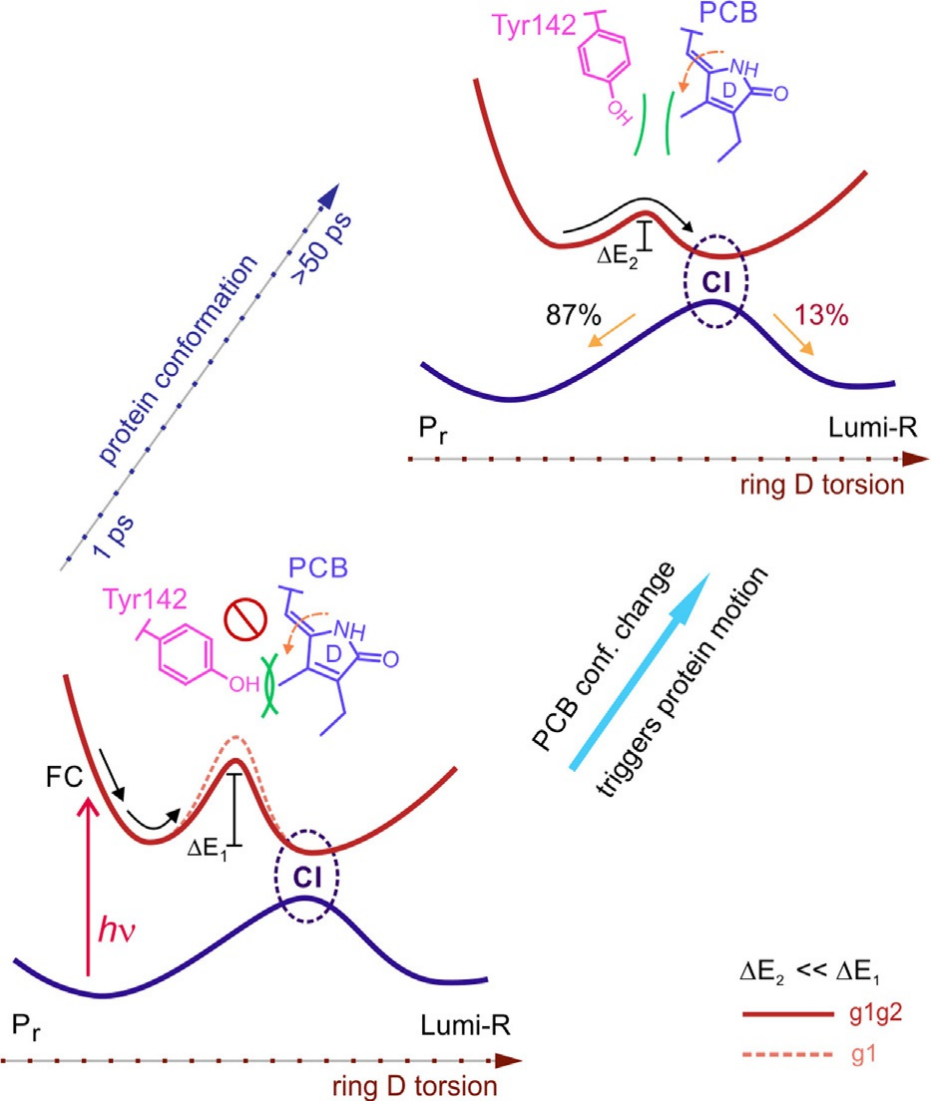
Main reaction coordinates determining the photoconversion kinetics in the P_r_ form of g1 and g1g2. For the larger construct, the interaction of the “tongue” region with the PCB chromophore results in a lower barrier (red, solid vs. orange, dashed lines). The early (<10 ps) dynamics of the PCB chromophore triggers reorganizations in the protein binding pocket, which lower the energetic barrier at later timescales (Δ*E*_1_ vs. Δ*E*_2_) and unlock the photoisomerization.

Based on the analysis presented above, we propose the following molecular picture for the photoisomerization dynamics of g1g2 (Figure 6). After excitation, the PCB chromophore leaves the FC region (∼100 fs) and undergoes ES conformational dynamics on the early ps timescale (<20 ps). This dynamics acts as a trigger for larger scale motions in the protein environment, which alleviates restrictions hindering further evolution on the ES (illustrated by the barrier on the ES potential energy surface). Interestingly, similar conclusions were derived in recent studies on related bacteriophytochromes[^51^, ^52^] and a cyanobacteriochrome.^[53]^ As the protein reorganizes, the barrier on the ES decreases which allows P_r_^*^ relaxation to proceed. This model explains the distributed character of the P_r_^*^ decay kinetics as it is imposed by the conformational dynamics of the protein. Eventually, P_r_^*^ decays (∼130 ps) yielding the primary photoproduct Lumi‐R.

Previously, we demonstrated that the ring D rotation of the PCB chromophore in g1 is hindered by a nearby Tyr residue (Tyr142).^[8]^ In the g1g2 (GAF‐PHY) construct, the interaction of the “tongue” region of the PHY domain with the chromophore‐binding pocket in the GAF domain limits the conformational space of rings A and D of the PCB and drives the Tyr142 residue away from the chromophore as compared with g1.^[32]^ Therefore, it appears as if the “tongue” region keeps the chromophore and the binding pocket in a more reactive conformation. This decreases the extent of protein conformation reorganization required for facilitating the isomerization of the PCB chromophore and results in an accelerated P_r_^*^ decay kinetics (Figure 5) and a more efficient P_r_→P_fr_ photoconversion (less energy being lost on nonproductive degrees of freedom).

## Conclusions

Our work provides direct evidence for the essential role of the protein environment in the control of the photoisomerization kinetics of the PCB chromophore and outlines a detailed mechanistic picture of the P_r_^*^ photoisomerization dynamics in knotless phytochromes. From an evolutionary perspective, the PHY domain “tongue” represents a development in phytochromes that tunes the photoreception efficiency. This is a key design principle for the development of optimized photoreceptors for biotechnological applications.

## Conflict of interest

The authors declare no conflict of interest.

## Supporting information

As a service to our authors and readers, this journal provides supporting information supplied by the authors. Such materials are peer reviewed and may be re‐organized for online delivery, but are not copy‐edited or typeset. Technical support issues arising from supporting information (other than missing files) should be addressed to the authors.

Supplementary
