# Supplementary material for: Effect of the PHY Domain on the Photoisomerization Step of the Forward Pr→Pfr Conversion of a Knotless Phytochrome
Source: Chemistry. 2020 Nov 27;26(71):17261–6. doi: 10.1002/chem.202003138 (PMC7839672; doi:10.1002/chem.202003138)
Supplement: Supplementary file 1 — Supplementary [file CHEM-26-17261-s001.pdf]

# Chemistry–A European Journal

Supporting Information


## Effect of the PHY Domain on the Photoisomerization Step of the Forward $P_r \longrightarrow P_{fr}$ Conversion of a Knotless Phytochrome


Tobias Fischer,[a] Qianzhao Xu,[b] Kai-Hong Zhao,[c] Wolfgang Gärtner,[b] Chavdar Slavov,*[a] and Josef Wachtveitl*[a]

## Materials and methods

### Stationary characterization

Absorption spectra of All2699g1g2 were recorded on a Specord S600 absorption spectrometer (Analytik Jena). To photoconvert the sample from the $P_r$ to the $P_{fr}$ state and reverse we used 590 nm (M590L3, Thorlabs) and 730 nm (M730L4, Thorlabs) LEDs, respectively. To avoid accumulation of photoproducts, the sample was photoconverted back to the desired state for each measurement. The pure $P_{fr}$ spectrum was generated by subtracting a scaled $P_r$ spectrum from the spectrum of the photostationary state reached after illumination at 590 nm ($PSS_{590}$). This scaling factor S was used to optimally remove the $P_r$ contribution. The obtained spectrum was then multiplied by 1+(S/(1-S)) to account for a complete conversion to the $P_{fr}$ state. The factor S was determined as 0.38 yielding a factor of 1.61 to account for complete conversion to the $P_{fr}$ state.

CD spectra were measured with a J-710 CD-spectrometer (Jasco) under a constant nitrogen flow of 2.0 l/min. Again, to avoid accumulation of photoproducts, samples were irradiated with the appropriate LED after each scan. Five single scans were recorded and averaged to yield the spectra of the $P_r$ state and the PSS. The pure $P_{fr}$ spectrum was obtained analogous to the UV-vis spectra by subtracting the $P_r$ spectrum scaled with a factor of 0.38 from the PSS spectrum. The obtained spectrum was then scaled by 1.61 to account for complete conversion to the $P_{fr}$ state.

Quantum yields (QY) were determined using a V-650 spectrometer (Jasco) by monitoring the induced absorption changes at 715 nm under constant illumination by the appropriate LED at a set temperature of 20 °C. The light was focused and recollimated into an optical fiber and led into the cuvette emerging directly above the sample surface. This ensures that the complete output of the fiber is illuminating the sample. The intensity of the light entering the sample cuvette was measured using a calibrated light detector (P-9710, Gigahertz-Optik). The changes in absorption were measured for 5 min every 2 s. In a separate measurement, the probe light was determined to have negligible actinic effect. For the QY calculations, only the linear part of the absorption changes (first 2 min) was used, as in this time period the product absorption is negligible and thus the absorption changes depend on the amount of absorbed light in a linear way. The calculations were performed as described previously.[1]

### Vis-pump-probe

The time-resolved transient absorption (TA) measurements were performed using a home-built pump–probe setup. This setup was described in detail in our previous work.[2] In short, the ultrashort laser fundamental pulses (100 fs, 800 nm) were provided by an Ti:Sa amplifier system (Spitfire Ace-100F-1K, Spectra-Physics) at a repetition rate of 1 kHz. The pump pulses for the experiments were generated in a home-built two stage NOPA (noncollinear optical parametric amplifier)[3,4] and were compressed using a prism compressor placed between the two NOPA stages. To generate the probe pulses the laser fundamental was focused into a $CaF_2$-crystal (5 mm). The signal was detected in reference mode by two spectrometers (Carl Zeiss, MMS UV/VIS II) equipped with 366 lines/mm gratings and a 256-channel photodiode array (Hamamatsu, S3904-

256Q). The instrument response function (IRF) in the experiments, estimated from the pump-probe cross-correlation, was ~80 fs. Anisotropic contributions were eliminated by measuring under magic angle conditions (54.7° pump-probe polarization difference). The experiments were conducted in a fused silica cuvette with 1 mm optical path length. The cuvette was continuously moved in the plane perpendicular to the direction of probe pulse propagation to avoid accumulation of photoproducts. To keep the sample in the $P_r$ state the cuvette was continuously irradiated with a high-power LED at 730 nm.

**Data analysis**

The analysis of the experimental data was performed using OPTIMUS (www.optimusfit.org).[5] Lifetime distribution analysis (LDA) was used for the ultrafast TA data as a model independent method of analysis that naturally deals with non-exponential or distributed kinetics. In this analysis, the pre-exponential amplitudes of a set of 100 exponential functions with fixed, equally spaced (on a decimal logarithm scale) lifetimes are determined. The obtained pre-exponential amplitudes at each detection wavelength can be presented in the form of a contour lifetime density map (LDM)[6]. The reading of the LDMs is as for a decay-associated spectrum from global lifetime analysis: (i) positive (red) amplitudes account for decay of excited state and product absorption (ESA, PA) or rise of ground state bleach and stimulated emission (GSB and SE); (ii) negative (blue) amplitudes account for rise of absorption (ESA, PA) or decay of GSB and SE. Global target modeling was performed as described previously.[5,7] The experimental data were fitted using a sequential kinetic scheme, which results in increasing lifetimes and evolution-associated difference spectra (EADS)[5,7]. The EADS can be interpreted as the spectral evolution of the recorded signals. The TA data around time zero position contain the so-called coherent artifact contributions. These were approximated with a function composed of a Gaussian and/or its first and second derivative[5,8] and fitted within the same routine as the exponential fitting.

**SI References**


[1] C. Slavov, N. Bellakbil, J. Wahl, K. Mayer, K. Rück-Braun, I. Burghardt, J. Wachtveitl, M. Braun, *Phys. Chem. Chem. Phys.* **2015**, *17*, 14045–14053.

[2] C. Slavov, T. Fischer, A. Barnoy, H. Shin, A. G. Rao, C. Wiebeler, X. Zeng, Y. Sun, Q. Xu, A. Gutt, K. Zhao, W. Gärtner, X. Yang, I. Schapiro, J. Wachtveitl, *Proc. Natl. Acad. Sci. U. S. A.* **2020**, *117*, 16356–16362.

[3] T. Wilhelm, J. Piel, E. Riedle, *Opt. Lett.* **1997**, *22*, 1494.

[4] E. Riedle, M. Beutter, S. Lochbrunner, J. Piel, S. Schenkl, S. Spörlein, W. Zinth, *Appl. Phys. B* **2000**, *71*, 457–465.

[5] C. Slavov, H. Hartmann, J. Wachtveitl, *Anal. Chem.* **2015**, *87*, 2328–2336.

[6] R. Croce, M. G. Müller, R. Bassi, A. R. Holzwarth, *Biophys. J.* **2001**, *80*, 901–915.

[7] I. H. M. M. van Stokkum, D. S. Larsen, R. van Grondelle, *Biochim. Biophys. Acta - Bioenerg.* **2004**, *1657*, 82–104.

[8] S. A. Kovalenko, A. L. Dobryakov, J. Ruthmann, N. P. Ernsting, *Phys. Rev. A - At. Mol. Opt. Phys.* **1999**, *59*, 2369–2384.

[9] M. G. Müller, I. Lindner, I. Martin, W. Gärtner, A. R. Holzwarth, *Biophys. J.* **2008**, *94*, 4370–4382.

[10] C. Slavov, X. Xu, K. H. Zhao, W. Gärtner, J. Wachtveitl, *Biochim. Biophys. Acta - Bioenerg.* **2015**, *1847*, 1335–1344.

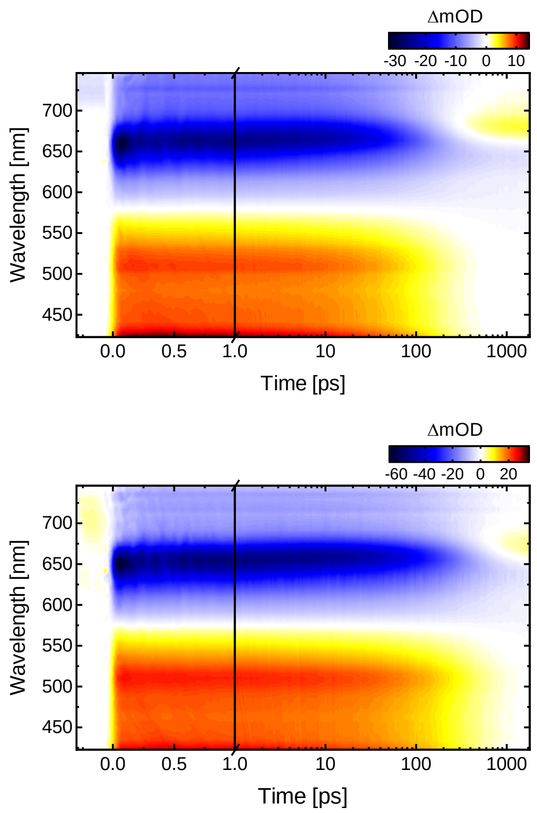


**Figure S1. TA data from the primary forward dynamics of All2699g1g2 (top) and All2699g1[2] (bottom).** TA data after excitation at 635 nm. Negative absorption difference signals (GSB and SE) in blue/black and positive absorption difference signals (ESA and PA) in yellow/red.

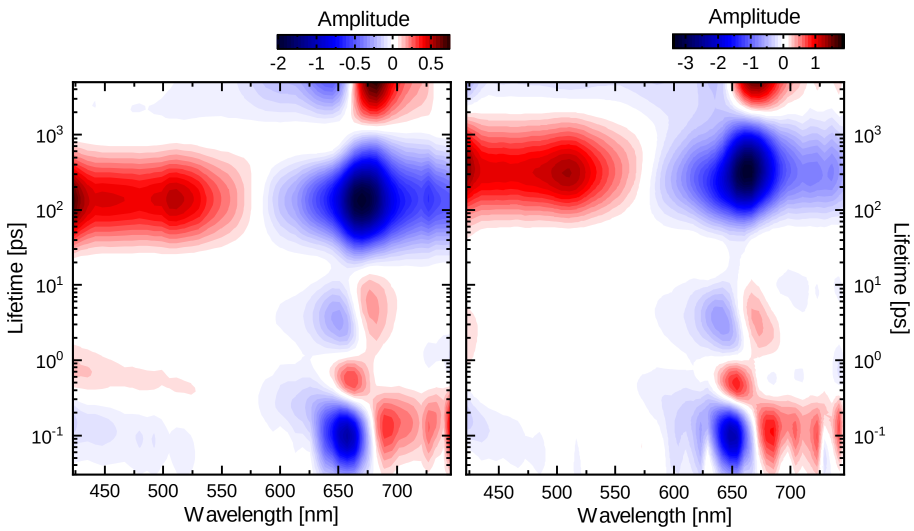


**Figure S2. Lifetime density maps (LDMs) of All2699g1g2 (left) and All2699g1[2] (right) obtained from the lifetime distribution analysis of the TA data shown in Fig. S1.** Positive amplitudes (red) account for decay of absorption (ESA, PA) or rise of GSB and SE, while negative amplitudes (blue) account for rise of absorption (ESA, PA) or decay of GSB and SE.

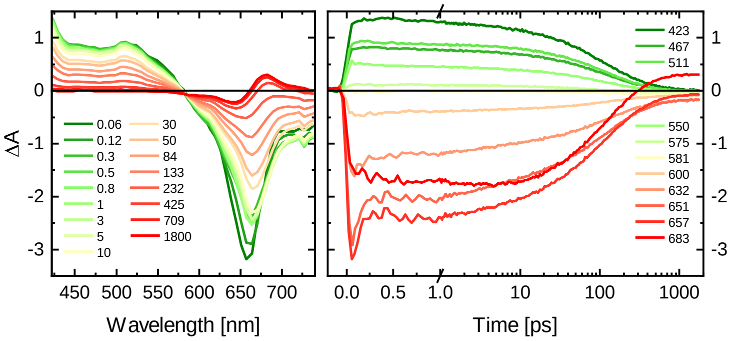


**Figure S3. TA data from the primary forward dynamics of All2699g1g2. Left:** Transient spectra at selected delay times (in ps). **Right:** transient decays at selected detection wavelengths (in nm). Positive signals correspond to excited state (ESA) and product absorption (PA), while negative signals correspond to ground state bleach (GSB) and stimulated emission (SE).

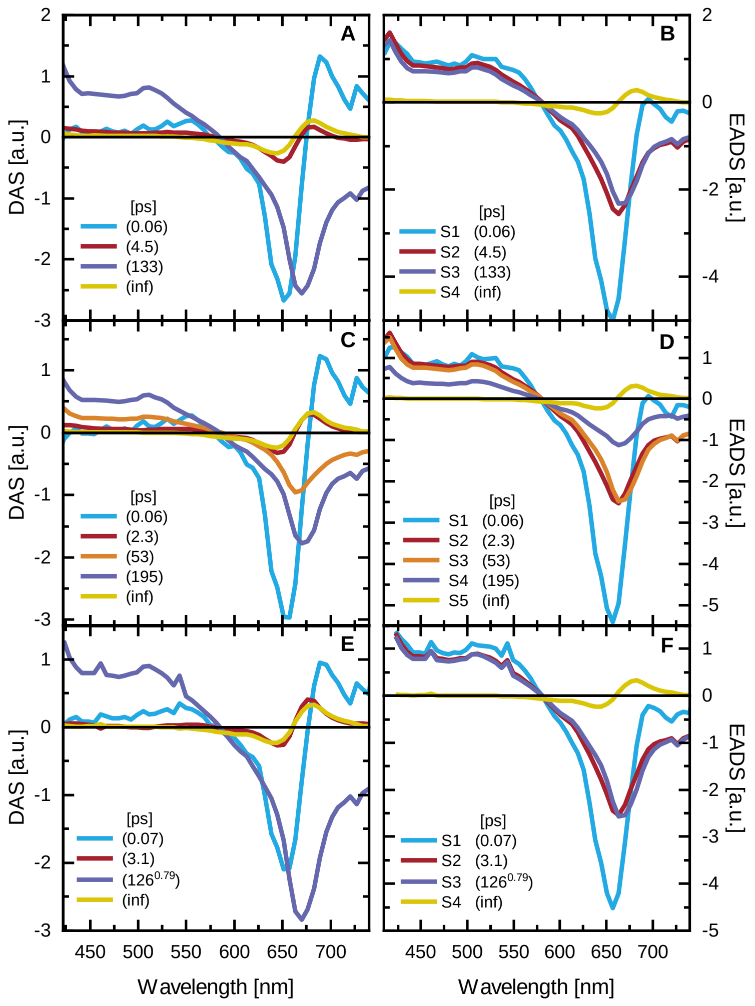


**Figure S4. Analysis of the TA dataset of All2699g1g2 using different sequential kinetic schemes.** Models with 4 states (A, B), five states (C, D) and 4 states, where the third state S3 was described by a stretched exponent (E, F) were used to fit the data. The kinetic model fitting results in the decay-associated spectra (left) and the evolution-associated difference spectra (EADS) (right). For a comparison of fit quality see Fig. S5.

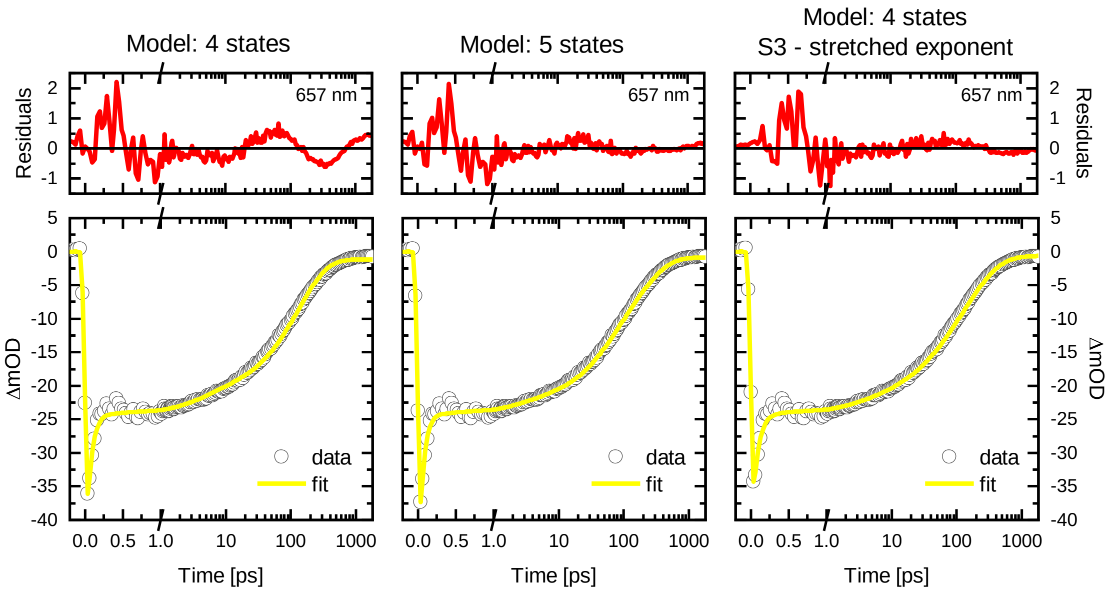


**Figure S5. Comparison of data fit quality for 3 different sequential models at 657 nm.** Note that independent of the model used for the data fitting, the residuals show strong high frequency oscillations in the 0-1 ps time range. These oscillations are present already in the experimental data (Fig S3) and are due to coherent oscillation in the transient absorption originating from coherent wavepacket motion on the excited state potential energy surface. Such coherent oscillations are often observed in the ultrafast transient absorption data of phytochromes[2,9,10]. Due to their high frequency character, the oscillations are not fitted by pure exponential functions and remain in the residuals. At later timescales (10 ps to 1.8 ns), however, the oscillation in the residuals represent poor fit quality for a given model. Therefore, it is evident that the ordinary four-state model (left) does not reproduce well the experimental data.
